# New Limonoids and a Dihydrobenzofuran Norlignan from the Roots of *Toona sinensis*

**DOI:** 10.3390/molecules18032840

**Published:** 2013-03-01

**Authors:** Xiao-Jie Dong, Yun-Fei Zhu, Guan-Hu Bao, Feng-Lin Hu, Guo-Wei Qin

**Affiliations:** 1 Key Laboratory of Tea Biochemistry and Biotechnology, School of Tea and Food Technology, Anhui Agricultural University, Hefei 230036, China; 2 Research Center on Entomogenous Fungi, School of Forestry, Anhui Agricultural University, Hefei 230036, Anhui, China; E-Mail: hufenglin@hotmail.com; 3 Department of Natural Products Chemistry, Shanghai Institute of Material Medica, Shanghai 201203, China; E-Mail: gwqin@mail.shcnc.ac.cn

**Keywords:** Meliaceae, *Toona sinensis*, limonoids, DPPH free radical scavenging

## Abstract

Two new limonoids, toonins A (**1**) and B (**2**), and one new dihydrobenzofuran norlignan, toonin C (**3**), were isolated from the roots of *Toona sinensis* together with the ten known compounds 4-methoxy-6-(2′,4′-dihydroxy-6′-methylphenyl)-pyran-2-one (**4**), bourjotinolone A (**5**), proceranone (**6**), matairesinol (**7**), 4-hydroxy-3-methoxybenzene-ethanol (**8**), syringic acid (**9**), isoscopoletin (**10**), lyoniresinol (**11**), aloeemodin (**12**), and *β*-sitosterol (**13**). Their structures were elucidated on the basis of one- and two-dimensional spectroscopic analysis. Isolation of compounds **4**, **6**–**13** from this plant is reported here for the first time.

## 1. Introduction

**Figure 1:**
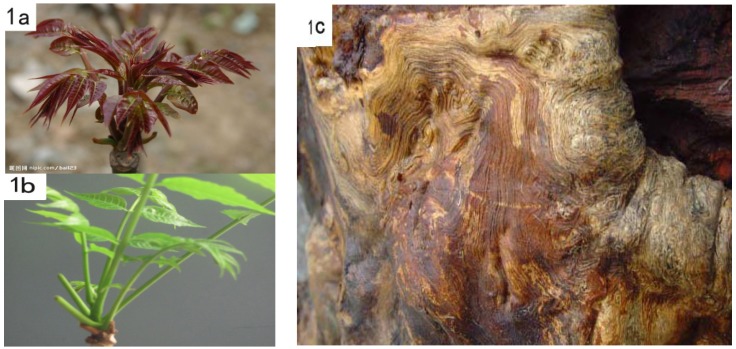
(**a**) Red young leaves of *Toona sinensis*; (**b**) Green young leaves of *Toona sinensis*; (**c**) Roots of *Toona sinensis*.

*Toona sinensis* (syn. Cedrela sinensis A. Juss, Meliaceae) is a woody plant native to eastern and southeastern Asia that has more than 2,000 years of cultivation history in China [1]. The various parts tissues of this species are widely used in Traditional Chinese Medicine (TCM). The leaves and stems of this plant were used for the treatment of itch, dysentery, and enteritis [2]. The bark was used as an astringent and depurative, the powdered roots were used as a corrective, and the fruits were used as an astringent and for the treatment of eye infections [3]. Previous phytochemical investigations on this species have resulted in the isolation of flavonoids, phenolics, alkaloids, terpenes, anthraquinones, and limonoids [4,5,6,7]. Besides its uses in TCM, the young leaves of *T. sinensis* (xiāngchūn) have been used extensively as a vegetable source in China for their special onion-like flavor and wealth of nutrients such as carotene and vitamins B and C, *etc.* The cultivated variety with red young leaves (Figure 1a) is considered of better flavour than those with green leaves (Figure 1b). Taihechun (a red variety originated from Taihe County, Anhui Province) is one of the most famous plants in this species, and was used for royal tributes during the Tang Dynasty [1]. The variety studied in this paper, also a red one, was collected from Tonglin County, where it is cultivated in the rocky mountainous regions by the local farmers. Interestingly, this variety very grows well, with very few pest incursions. A possible reason is that specific pest-repellent constituents may exist in the roots (Figure 1c). To our knowledge, no previous chemical studies have been published on the roots of this variety. To further search for the novel bioactive agents from Meliaceae plants, the roots of *T. sinensis* were investigated guided by LC-PAD-MS analytical data together with DPPH assay [8]. The purification work was mostly carried out employing two preparative HPLC separations after a silica gel column fractionation. Thirteen compounds, including limonoids, phenols, and other compounds were isolated and identified*.*

## 2. Results and Discussion

**Figure 2:**
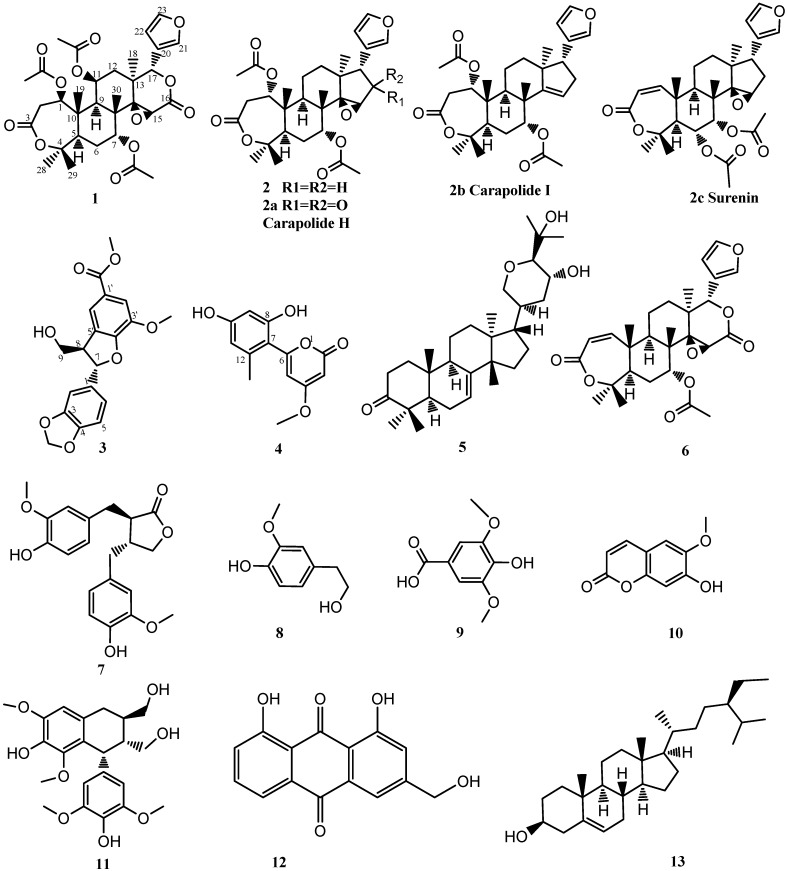
Structures of the compounds **1**–**13**.

The EtOH extract of the roots of *T. sinensis* was suspended in 70% aqueous MeOH and extracted by CHCl_3_. The CHCl_3_-soluble fraction was subjected to silica gel column chromatography and repeated RP-18 HPLC to yield two new limonoids **1** and **2**, and one new phenylpropanoid **3**, as well as ten known compounds, which were identified as 4-methoxy-6-(2′,4′-dihydroxy-6′-methylphenyl)- pyran-2-one (**4**) [9], bourjotinolone A (**5**) [10], proceranone (**6**) [11], matairesinol (**7**) [12], 4-hydroxy-3-methoxybenzeneethanol (**8**) [13], syringic acid (**9**) [14], isoscopoletin (**10**) [15], lyoniresinol (**11**) [16], aloeemodin (**12**) [17], and *β*-sitosterol (**13**), respectively, on the basis of their spectroscopic data and by comparison with reference data (Figure 2).

**Figure 3:**
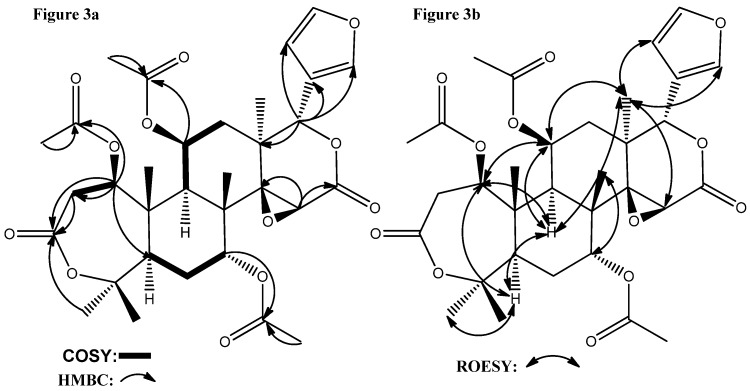
(**a**) Selected HMBC and ^1^H-^1^H COSY correlations for **1**; (**b**) Selected ROESY correlations for **1**.

Compound **1** was obtained as an amorphous white powder (CH_3_OH). The molecular formula was established as C_32_H_40_O_12_ by HRESIMS (*m/z* 639.2411 [M+Na]^+^, calcd. for C_32_H_40_O_12_ + Na; 634.2860 [M+NH_4_]^+^ calcd. for C_32_H_40_O_12_ + NH_4_) in positive mode. Its IR bands at 1741, 1720, 1600, 1504 cm^−1^ indicated the existence of carbonyl groups and olefinic carbons. Its NMR spectra (Table 1) generally resembled those of nomilins such as 11*β*,19-diacetoxy-l-deacetyl-l-epidihydronomilin [18], 7-acetyl-11*β*-acetoxy-dihydronomilin [19] and 11β-hydroxycneorin G [20], suggesting that **1** had a limonoid skeleton. The ^1^H-NMR spectrum indicated the presence of five tertiary methyls (five sharp single peaks at *δ*: 1.20, 1.34, 1.37, 1.44, 1.49), three acetate methyl groups (three single peaks at *δ*: 2.05, 2.08, 2.13) and three oxymethine protons (*δ*: 4.83, 4.51, 5.16), and an α-oriented furan ring (*δ*: 6.28, 7.37, and 7.41, 1H each). The ^13^C-NMR spectrum indicated the presence of five carbonyl carbons, including three acetate groups (*δ*: 170.00, 169.95, 169.20) and two lactone moieties (*δ*: 169.98, 166.58). In the HMBC spectrum (Figure 3a), the cross-signal between *δ*_C_ 170.00 and *δ*_H_ 4.83 (H-1) demonstrated that the acetoxy group was attached to C-1. The HMBC data also indicated that the acetoxy group (*δ*_C_ 169.95) was attached to C-7 (*δ*_H_ 4.51) and the one at *δ*_C_ 169.20 was attached at C-11 (*δ*_H_ 5.16). The correlations of the carbonyl C-3 resonance at *δ* 169.98 to the proton signals of H-2 and CH_3_-28 (a four-bond correlation) suggested that the A-ring of **1** was a lactone moiety. The cross-signals between H-15/C-14, H-15/C-16, H-17/C-13, and H17/C-20 suggested the presence of a δ-lactone moiety with a 14,15-epoxide (*δ*_H-15_ 3.55s, *δ*_C-15_ 5.70) in the D-ring of **1**.

As regards the relative stereochemistry of **1**, The ROESY correlations (Figure 3b) detected between H-5/H-9, H-5/H3-28, H-9/H-11, H-9/H3-18, H3-18/H-11, H3-18/H-15, H3-18/H-21, H3-18/H-22, and H3-30/H-7 showed that H-5, OAc-7, H-9, H-11, Me-18, and Me-28 were in an α-orientation, whereas OAc-11, the 14,15-epoxide, H-17, Me-19, Me-29, and Me-30 were in a β-orientation, as in 11*β*-hydroxycneorin G [20]. Finally, the ROESY correlations between H-1/H-5, H-1/H-9, H-1/H-11, which showed strong similarity to the case of 11*β*,19-diacetoxy-l-deacetyl-l-epidihydronomilin [18], clearly decided the α-orientation of H-1 and the β-orientation of OAc-1. In the ROESY spectrum, the most easily confused signals are the overlapped ones of Me-19 and H-12*β*, both of which are at *δ* 1.44, in which case the related ROESY cross-signals cannot be used for the corresponding relative stereochemistry determination. However, when the Me-19 is oxygenated, its chemical downshifts and H-12*β* is a distinct dd peak at around *δ* 1.41 [18], which can be used for the relative stereochemistry determination. Accordingly, the structure of **1** was determined to be 7-acetyl-11*β*-acetoxy-l-epidihydronomilin as shown in Figure 2, and it was named toonin A. 

**Table 1:** ^1^H- and ^13^C-NMR spectral data (δ in ppm, *J* in Hz) of **1** and **2** in CDCl_3_^a^.

Toonin A (1)					Toonin B (2)	
Atom	Proton	*J* (Hz)	Carbon	HMBC (H-C)	Proton	*J* (Hz)
1	4.83 d	7.0	71.12	C-2, 3, 5, 1-OCOCH_3_	4.94 d	7.0
2α	3.14 d	17.1	34.58	C-1, 3, 10	3.12 dd	17.1, 7.0
2β	3.22 dd	17.1, 7.0			3.21 d	17.1
3			169.98			
4			84.42			
5	2.43 brd	13.5	44.51	C-4, 6, 10, 19, 29	2.52 dd	13.5, 2.0
6α	2.12 m	13.5	26.08		1.99 brd	13.5
6β	1.88 brt				1.84 m	
7	4.51 brs		74.39	C-5, 9, 24	4.70 brs	
8			41.65			
9	2.88 d	3.0	40.00	C-8, 10, 19, 30	2.91 dd	11.5, 2.0
10			45.75			
11	5.16 m		67.24		1.55, 1.62 m	
12α	2.29 dd	15.7, 11.5	37.02	C-9, 14	1.84, 1.62 m	
12β	1.44 m					
13			37.87			
14			68.61			
15	3.55 s		54.70	C-14, 16	3.42 s	
16			166.58			
17	5.57 s		78.32	C-12, 14, 18, 21, 22, 23	2.64 m	
18	1.20 s		16.88	C-12, 13, 14, 17	0.94 s	
19	1.44 s		17.10	C-1, 5, 9	1.18 s	
30	1.34 s		20.00	C-7, 8, 9, 14	1.11 s	
28	1.49 s		34.53	C-4, 5, 29	1.51 s	
29	1.37 s		23.41	C-4, 5, 28	1.40 s	
7-OCOCH_3_			169.95			
7-OCOCH_3_	2.08		21.57	7-OCOCH_3_	2.07 s	
1-OCOCH_3_			170.00			
1-OCOCH_3_	2.13		21.03	1-OCOCH_3_	2.16 s	
11-OCOCH_3_			169.20			
11-OCOCH_3_	2.05		20.63	11-OCOCH_3_		
21	7.37		140.80	C-20, 22, 23	7.11 s	
20			120.00			
22	6.28		109.82	C-21, 20, 23	6.15 s	
23	7.41		142.92	C-21, 20	7.38 s	

*^a^* ^1^H-NMR recorded at 500 MHz and ^13^C-NMR at 1205 MHz.

Compound **2** was obtained as an amorphous white powder (CH_3_OH). The molecular formula was established as C_30_H_40_O_8_ by HR-ESIMS at *m/z* 551.26166 [M+Na]^+^ (calcd. for C_30_H_40_O_8_ + Na), 567.23561 [M+K]^+^ (calcd. for C_30_H_40_O_8_ + K) in positive mode. Its NMR spectra generally resembled compound **1** and showed strong similarity to the evodulane type nomilins carapolide H and carapolide I (**2a**,**2b**, Figure 2) [21]. The ^1^H-NMR spectrum revealed resonances for five tertiary methyl groups, two acetoxy groups, and an epoxide proton (s, *δ*: 3.42, H-15). In addition to the characteristic α-orientation furan signals, two oxymethine resonances were clearly discernable at *δ*: 4.94 (d, *J* = 7.0 Hz, H-l) and 4.70 (brs, H-7). The HMBC cross-signals can deduce the OAc groups were attached at C-1 and C-7. The correlations of the carbonyl C-3 resonance at *δ* 170.00 to the proton signals of H-1 and CH_3_-28 suggested the existence of a lactone moiety at A-ring of **2**. Compared with carapolide H (**2a**, Figure 2), the D-ring of compound **2** has the epoxide signal (s, *δ*_H-15_: 3.42s; *δ*_C-15_: 56.73), however, it is a CH_2_ signal of C-16 (*δ*_H-16_: 1.65, 2.71, *δ*_C-16_: 31.54) instead of a ketone carbon. Through further literature research, the NMR data of the D-ring was found to be very similar to the D-ring of surenin (**2c**, Figure 2) [22] whose C-16 is also a methylene**.** The correlations at H2-16/C-13, H2-16/C-17, H-17/C-13, H-17/C-18, H-17/C-20, decided all the D-ring signals (*δ*_H-16_: 1.65, 2.71, *δ*_C-16_: 31.54; *δ*_H-17_: 2.64, *δ*_C-17_: 39.16). And H-17/C-20 (*δ*_C-20_: 123.03), H-17/C-21(*δ*_C-21_: 138.84) revealed the furan ring [7.11 (1H, s, H-21), 6.15 (1H, s, H-22), 7.38 (1H, s, H-23); 123.03 (C-20), 138.84(C-21), 110.92 (C-22), 142.71 (C-23)] was attached at C-17. Therefore, structure **2** was attributed to 14,15-epoxycarapolide I and named toonin B.

Compound **3** was obtained as an amorphous white powder (CH_3_OH). The molecular formula C_19_H_18_O_7_ was established by HR-ESIMS^+^: *m/z* 359.1125 [M+H]^+^ (calcd. for C_19_H_18_O_7_ + H), 381.0945 [M+Na]^+^, 739.1997 [2M+Na]^+^ in positive mode. Its IR spectrum showed characteristic absorptions for hydroxyl group (3445 cm^−1^), carbonyl group (1712 cm^−1^), and phenyl group (1613, 1599, 1492 cm^−1^). The NMR data was very similar to that of the known compound cedralin A [6]. The only difference, one more methoxyl group at [*δ*_H_ 3.81 (3H, s, OCOCH_3_-1′), *δ*_C_ 51.92 (OCOCH_3_-1′)] of **3**, suggested methyl esterification of the carboxylic acid group at C-1′ of cedralin A [6] leading to production of **3**, so compound **3** was identified as a (7*S*,8*R*)-7,8-dihydro-8-(hydroxymethyl)-3′-methoxy-7-[3,4-(methylenedioxy) phenyl] benzofuran-1′-carboxylic acid methyl ester) and named toonin C (Figure 2).

Compound **4**, 4-methoxy-6-(2′,4′-dihydroxy-6′-methylphenyl)-pyran-2-one, had previously been reported [9,23,24,25] and its structure had been confirmed by X-ray analysis [24] and ^1^H-NMR spectra reported by Conner in 1987 [25]. However, its ^13^C-NMR spectrum has never been provided, so this paper gives for the first time a detailed description of its ^13^C-NMR spectrum.

The DPPH assay showed that the EtOH extract of the root was more active than that of the leaves (Table 2). The CHCl_3_ fraction from the roots was also very active.

**Table 2:** Free Radical Scavenging Capacity of the extracts.

Tested samples	Ascorbic acid	EtOH extract of leaves	EtOH extract of roots	CHCl_3_ fraction from roots
	(0.05 mM)	(0.25 mg/mL)	(0.25 mg/mL)	(0.25 mg/mL)
Clearing ratio	59.5 ± 0.8	86.5 ± 1.6	91.1 ± 1.2	80.5 ± 1.4

Our research showed that the extract of *T. sinensis* had certain antioxidant capacity, which gave substantial support for its historical use as a healthy food. Our continuing chemical investigation on the free radical scavenging active fraction of the roots of *T. sinensis* yielded three new and ten known compounds, among which eight known phenols (**3**, **4**, **7**–**12**) and four Meliaceae limonoids (**1**, **2**, **5**, **6**) were found. 

Limonoids **1** and **6** belong to the ring A,D-seco group, and compound **2** is a ring A-seco group compound, while compound **5** without a seco-ring is a protolimonoid [26]. The ring A,D-seco and the protolimonoid groups have been isolated from the leaves, seeds, stems, or cortex of *T. sinensis*. The ring A,D-seco group with a functional oxygen group at position 11 (C-11) has been used as the distinguishing feature for species of Meliaceae [20]. However, besides the C-11 oxygenated ones, our research showed that C-11 non-oxygenated limonoids also exist in this plant. Compound **2** belongs to the evodulone type of limonoids, featuring a ring A lactone. These types of limonoids are examples of an A ring opened before the D ring, which are not common in limonoids from the Meliaceae family.

## 3. Experimental

### 3.1. General

All solvents used in the extraction and isolation processes were redistilled prior to use. IR spectra (KBr) were obtained on a Perkin Elmer Spectrum 2000 instrument. All NMR spectra were recorded on a Bruker DRX-500 instrument in CDCl_3_ or DMSO-*d*_6_. Chemical shifts are reported in ppm using TMS as internal standard. High-resolution ESI mass spectra were obtained on a JEOL-LC Mate LCMS system. LC-MS analyses were carried out using an Esquire 3000 instrument equipped with autosampler and DAD. Bruker DataAnalysis 3.1 software was used for data acquisition and processing. A Lichrocart cartridge, set 55-2, Merck 1.5024 was used as separation column, flow rate was 0.5 mL/min, time 15.0 min. Gradient elution was performed with water/0.05% formic acid (solvent A) and acetonitrile/0.05% formic acid (solvent B) at a constant flow rate of 0.5 mL/min. An increasing linear gradient (v/v) of solvent B was used [*t* (min), %B]: 0, 0; 9.5, 100; 12.2 100; 12.3, 0; 15.0, 0. Detection was carried out at 200 nm, with peak scanning between 195-600.2 nm. All of the analyses were carried out using a Turbo Ionspray source in the negative mode with the following settings: capillary exit, 100 V; nebulizer, curtain, collision, and drying gas (N_2_). Full scan acquisition was performed by scanning from *m/z* 100 to 1,750 U in profile at a cycle time of 2 s with a step size of 0.1 U and a pause between each scan of 2 ms. MS/MS product ions were produced by collision-activated dissociation (CAD) of the selected precursor ions in the collision cell of the triple quadrupole mass spectrometer and analyzed using the second analyzer of the instrument. In all of the experiments, quadrupoles (Q1 and Q3) were operated at unit resolution. Different MS/MS experiments, neutral loss scan, product ion scan, and precursor ion scan of selected molecules were carried out to confirm the structure of the compounds previously identified by full scan mode. Analytical HPLC was performed on a Purospher STAR RP-18 column, 3 μm, 55 mm length × 4 mm ID, Gradient elution was performed with water/0.01% TFA (solvent A) and acetonitrile/0.01% TFA (solvent B) at a constant flow rate of 1.5 mL/min. An increasing linear gradient (v/v) of solvent B was used [*t* (min), %B]: 0, 10; 7.5, 100; 10, 100; 10.5, 10; 13.0, 10. Detection was carried out at 200 and 220 nm, with peak scanning between 195-600.2 nm. Millennium software was used for data acquisition and processing. An Agilent ChemicalStation was used off-line for library building and RT-UV library search. Preparative HPLC (Varian) for fractions 18 and 6 was performed on a Purospher RP-18 column, 12 μm, 250 mm length × 50 mm ID, Gradient elution was performed with water (solvent A) and acetonitrile (solvent B) at a constant flow rate of 40 mL/min. An increasing linear gradient (v/v) of solvent B was used [*t* (min), %B]: 0, 20; 65, 85; 80, 100; 95, 100. Detection was carried out at 210 nm. Preparative HPLC (Varian) for the 16th fraction: Purospher RP-18 column, 12 μm, 250 mm length × 25 mm ID, Gradient elution was performed with water (solvent A) and acetonitrile (solvent B) at a constant flow rate of 10 mL/min. An increasing linear gradient (v/v) of solvent B was used [*t* (min), %B]: 0, 15; 60, 45; 90, 100; 111, 100. Detection was carried out at 270 nm. Preparative HPLC (Varian) for the 30–39th fractions: Purospher RP-18 column, 12 μm, 250 mm length × 25 mm ID, Gradient elution was performed with water (solvent A) and acetonitrile (solvent B) at a constant flow rate of 10 mL/min. An increasing linear gradient (v/v) of solvent B was used [*t* (min), %B]: 0, 30; 60, 62; 90, 100; 111, 100. Detection was carried out at 250 nm.

*Plant Material.* The roots of *T. sinensis* were initially collected in Tonglin County of Anhui Province in October, 2011, and identified by one of the authors (G.-H.B.). A voucher specimen (No. AHAU2011102-1) was deposited in the herbarium of Anhui Agricultural University.

### 3.2. Compound Isolation

The air-dried and powdered roots (15 kg) were extracted with 95% EtOH (3 × 30 L) at room temperature. After removal of the solvent by evaporation under vacuum, the residue was diluted with 70% aqueous methanol (4 L) and then extracted successively with CHCl_3_ (4 × 4 L), EtOAc (3 × 4 L), and *n*-BuOH (4 × 4 L). The CHCl_3_ extract (355 g) was fractionated on a silica gel (100–200 mesh) column (11 × 100 cm) and gradually eluted by hexane-dichloromethane-methanol to yield 18 fractions. All of the 18 fractions were examined by TLC and LC-PAD-MS and some interesting constituents were found in fractions 6 (2.0 g), 14 (7.9 g), 15 (7.1 g), 16 (8.3 g) and fraction 18 (5 g). These five fractions were further fractionated by repeated preparative HPLC. Further preparative HPLC of Fraction 15 gave three limonoids: the new compounds toonin A (**1**, 14.5 mg, Rt 6.588 min), toonin B (**2**, 5 mg, Rt 7.256 min), and the known one proceranone (**6**, 10 mg, Rt 7.080 min). The new compound toonin C (**3**, 6.39 mg, Rt 7.256 min) and the known compound lyoniresinol (**11**, 10.3 mg, Rt 3.931 min) were obtained from Fraction 6. Further HPLC purification of fraction 18 yielded 4-methoxy-6-(2′,4′-dihydroxy-6′-methylphenyl)-pyran-2-one (**4**, 1.96 mg, Rt 4.577 min) and syringic acid (**9**, 5.68 mg, Rt 1.565 min). Fraction 14 was subjected to repeated preparative HPLC yielding bourjotinolone A (**5**, 82.6 mg, Rt 9.073 min) and matairesinol (**7**, 13.95 mg, Rt 5.368 min). 4-Hydroxy-3-methoxybenzeneethanol (**8**, 5.5 mg, Rt 0.974 min), isoscopoletin (**10**, 6.0 mg, Rt 3.716 min), aloeemodin (**12**), and *β*-sitosterol (**13**) were obtained by repeated HPLC purification of Fraction 16. 

### 3.3. Isolates

*Toonin A* (**1**). White amorphous powder, [α]_D_^25^ −25.3 (c 0.8, CHCl_3_). IR (KBr) ν_max_ (cm^−1^): 2987, 1741, 1720, 1508, 1444, 1370, 1221, 1123, 1024, 876, 774. ESIMS^−^: *m/z* 615.3 [M−H]^−^ (6), 677.2 [M+HCO_3_]^−^ (38), 156.9 (100) in negative mode, ESIMS^+^: *m/z* 639.2 [M+Na]^+^ (100) in positive mode; HRESIMS^+^: *m/z* 639.2411 [M+Na]^+^ (calcd. for C_32_H_40_O_12_ + Na), 634.2860 [M+NH_4_]^+^ in positive mode. ^1^H and ^13^C-NMR data (CDCl_3_), see Table 1.

*Toonin B* (**2**). White powder, [α]_D_^25^ −43.7 (c 0.8, CHCl_3_). IR (KBr) ν_max_ (cm^−1^): 2984, 2948, 1802, 1731, 1600, 1504, 1459, 1435, 1374, 1235, 1124, 1027, 874, 789 cm^−1^; EIMS: *m/z* 528 [M]^+^ (16), 468 (5), 410 (7), 350 (8), 208 (24), 107 (100), 79 (13), 43 (51); ESIMS^−^: *m/z* 527.2 [M−H]^−^ (94) in negative mode; ESIMS^+^: *m/z* 529.2 [M+H]^+^ (30), 546.2 [M+NH_4_]^+^ (100), 1074.1 [2M+NH4]^+^ (15) in positive mode; HRESIMS^+^: *m/z* 551.26166 [M+Na]^+^ (calcd. for C_30_H_40_O_8_ + Na), 567.23561 [M+K]^+^ in positive mode. ^1^H-NMR (CDCl_3_), see Table 1. ^13^C-NMR (CDCl_3_) *δ*: 70.48 (C-1), 34.08 (C-2), 170.00 (C-3), 85.24 (C-4), 44.08 (C-5), 26.47 (C-6), 73.22 (C-7), 41.73 (C-8), 37.05 (C-9), 44.25 (C-10), 15.80 (C-11), 29.42 (C-12), 41.46 (C-13), 72.48 (C-14), 56.73 (C-15), 31.54 (C-16), 39.16 (C-17), 21.02 (C-18), 15.82 (C-19), 123.03 (C-20), 138.84(C-21), 110.92 (C-22), 142.71 (C-23), 34.03 (C-28), 23.01 (C-29), 18.85 (C-30), 169.96 (OCOCH_3_-1), 21.07 (OCOCH_3_-1), 169.54 (OCOCH_3_-7), 20.60 (OCOCH_3_-7).

*Toonin C* (**3**). white powder (CH_3_OH); [α]_D_^25^ −22.6 (c 0.1, CH_3_OH); IR (KBr) *ν*_max_ (cm^−1^): 3445, 2948, 1712, 1613, 1599, 1492, 1435, 1331, 1250, 1108, 1038, 936, 769 cm^−1^; ESIMS^+^: *m/z* 359.2 [M+H]^+^ (52), 381.1 [M+Na]^+^ (100), 739.0 [2M+Na]^+^ (26); in positive mode; HRESIMS^+^: *m/z* 359.1125 [M+H]^+^ (calcd. for C_19_H_18_O_7_ + H), 381.0945 [M+Na]^+^, 739.1997 [2M+Na]^+^ in positive mode. ^1^H-NMR (DMSO-*d*_6_) *δ*: 6.88 (1H, d, *J* = 1.5 Hz, H-2), 6.91 (1H, d, *J* = 8.0 Hz, H-5), 6.84 (1H, dd, *J* = 8.0, 1.5 Hz, H-6), 5.60 (1H, d, *J* = 6.5 Hz, H-7*β*), 3.49 (1H, dd, *J* = 6.5, 5.5 Hz, H-8*α*), 3.69 (2H, m, H-9), 5.11 (1H, t, *J* = 7.0 Hz, HO-9), 7.44 (1H, s, H-2′), 7.55 (1H, s, H-6′), 6.01 (2H, s, OCH_2_O), 3.81 (3H, s, OCOCH_3_-1′), 3.83 (3H, s, OCH_3_-3′). ^13^C-NMR (DMSO-*d*_6_) *δ*: 134.94 (C-1), 106.17 (C-2), 146.99 (C-3), 148.36 (C-4), 108.36 (C-5), 119.30 (C-6), 86.67 (C-7), 52.45 (C-8), 62.58 (C-9), 121.94 (C-1′), 113.35 (C-2′), 142.66 (C-3′), 151.72 (C-4′), 129.44 (C-5′), 118.93 (C-6′), 100.98 (OCH_2_O), 166.30 (OCOCH_3_-1′), 51.92 (OCOCH_3_-1′), 56.08 (OCH_3_-3′). 

*4-Methoxy-6-(2′,4′-dihydroxy-6′-methylphenyl)-pyran-2-one* (**4**). White powder (CH_3_OH); IR (KBr) ν_max_ (cm^−1^): 3335, 2942, 1680, 1610 1558, 1514, 1454, 1406, 1336, 1269, 1222, 1161, 840. ESIMS^−^: *m/z* 247.1 [M−H]^−^ (100), 495.1 1 [2M−H]^−^ (5) in negative mode; ESIMS^+^: *m/z* 249.1 [M+H]^+^ (100), 271.1 [M+Na]^+^ (12), 497.1 [2M+H]^+^ (3) in positive mode; HRESIMS^−^: *m/z* 247.06118 [M−H]^−^ (calcd. for C_13_H_12_O_5_-H) in negative mode. ^1^H-NMR (DMSO-*d*_6_) *δ*: 5.60 (1H, d, *J* = 2.2 Hz, H-3), 6.12 (1H, d, *J* = 2.2 Hz, H-5), 6.22 (1H, d, *J* = 1.5 Hz, H-3′), 6.15 (1H, d, *J* = 1.5 Hz, H-5′), 3.83 (3H, s, OCH_3_-4), 9.55, 9.66 (2H, brs, HO-4, 5), 2.08 (3H, s, CH_3_-6′). ^13^C-NMR (DMSO-*d*_6_) *δ*: 164.90 (C-2), 88.62 (C-3), 171.74 (C-4), 105.34 (C-5), 159.30 (C-6), 111.57 (C-1′), 157.68 (C-2′), 99.96 (C-3′), 159.91 (C-4′), 109.24 (C-5′), 139.12 (C-6′), 56.28 (OCH_3_-4), 19.75 (CH_3_-6′). 

The assignments were confirmed by a combination of ^1^H-^1^H COSY, HSQC, HMBC, and ROESY spectra.

## 4. Conclusions

The chemical investigation on the free radical scavenging active fraction of the roots of *T. sinensis* yielded three new and ten known compounds, among which eight known phenols and four Meliaceae limonoids were found. The considerable number of phenols in the plants may account for its strong free radical scavenging activity. Isolation of compounds **4**, **6**–**13** from this plant is reported here for the first time.
